# Ab Initio Molecular Dynamics Simulation Study on the Molten Structure of Cryolite to the Cryolitic Bath, Na_3_AlF_6_–AlF_3_–Al_2_O_3_–CaF_2_

**DOI:** 10.1021/acsomega.5c05781

**Published:** 2025-09-05

**Authors:** Konstantinos Betsis, Konstantinos Karalis, Anthimos Xenidis

**Affiliations:** † School of Mining Engineering and Metallurgy, National Technical University of Athens, Zografou Campus, Athens 15780, Greece; ‡ University of Bern, Institute of Geological Sciences, Hochschulstrasse 6, Bern 3012, Switzerland

## Abstract

This study investigates the structural properties of cryolitic melts using ab initio molecular dynamics (AIMD) simulations. Aluminum production relies on the electrolysis of alumina in a molten cryolite bath, which primarily consists of cryolite, aluminum fluoride, alumina, and calcium fluoride and operates at temperatures between 1213 and 1243 Κ. Despite significant advancements, the local structure and speciation within these melts remain incompletely understood. This research employs computational techniques to examine the atomic structure and charge distribution in cryolitic melts, with a particular focus on aluminum atom interactions and the role of bridging anions. AIMD simulations were performed by using the CP2K software package. The Perdew–Burke–Ernzerhof (PBE) approximation was applied for the exchange-correlation functional, and Goedecker–Teter–Hutter (GTH) pseudopotentials were used to model core electrons. The study investigated systems with varying amounts of AlF_3_, Al_2_O_3_, and CaF_2_ in molten cryolite, maintaining temperatures slightly above the liquidus point. Structural analysis was conducted using radial distribution functions (RDFs) to determine bond distances and coordination numbers, while electronic distribution was analyzed through Mulliken population analysis. Key findings include the dominance of the AlF_5_^2–^ complex in molten cryolite, which is in agreement with previous studies. The addition of alumina influences the formation of oxyfluoroaluminate species, with Al_2_OF_6_^2–^ and Al_2_O_2_F_4_^2–^ being prevalent at low and high alumina concentrations, respectively. Calcium fluoride impacts the melt’s structure by increasing the presence of AlF_5_^2–^ and altering molecular conformation due to the strong anionic nature of calcium. The electronic structure analysis revealed minor changes in the average charge of atoms but an overall increase in the anionic character of the melt with the addition of O^2–^ and Ca^2+^. This study provides valuable insights into the atomic and electronic behavior of cryolitic melts, contributing to a deeper understanding of these complex molten systems and supporting the optimization of aluminum production processes.

## Graphical abstract

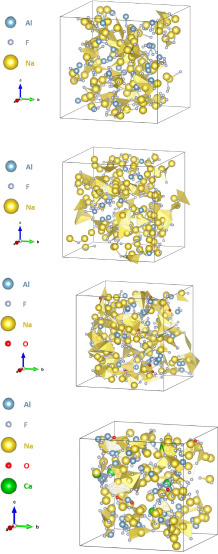

## 1 Introduction

Aluminum production is based on the reduction of alumina to molten aluminum via electrolysis. The electrolytic bath, consisting primarily of cryolite, aluminum fluoride, alumina, and calcium fluoride, operates in a temperature range of 1213–1243 Κ, with the specific operational temperature influenced by the chemical composition of the melt.^1^ Physicochemical properties such as density, viscosity, and electrical conductivity are influenced by structural speciation within the melt, which is investigated using both experimental techniques^2^ (Raman spectroscopy and nuclear magnetic resonance (NMR)) and theoretical computational methods. Despite substantial progress, our understanding of the local structure and the role of the main components within the cryolitic bath remains incomplete.

Numerous investigations have aimed to elucidate speciation in the Hall–Héroult electrolyte. Using Raman spectroscopic measurements, Gilbert et al.^3–5^ proposed the existence of three main aluminum complexes in the cryolite melt: AlF_4_^–^, AlF_5_^2–^, and AlF_6_^3–^. Their findings indicate that AlF_5_^2–^ is the dominant complex in molten Na_3_AlF_6_. These results are consistent with NMR experiments conducted by Cikit et al.,^6–9^ which also highlight the predominance of specific aluminum-containing molecules in the melt. Dewing^10^ suggested the coexistence of AlF_4_^–^, AlF_5_^2–^, and AlF_6_^3–^, as proposed by a thermodynamic data model. Ab initio molecular dynamics (AIMD) simulations have been used by several researchers^11–15^ to gain deeper insight into the local structure of molten cryolite and to quantify aluminum fluoride complexes in the melt. These simulations show that Na^+^ ions remain uncoordinated and free in the melt. The results reaffirm that AlF_5_^2–^ is the dominant complex in the melt, with either AlF_4_^–^ or AlF_6_^3–^ being the second most abundant complex in the melt.

The addition of alumina to molten cryolite influences the melt structure, depending on its concentration. Oxygen serves as a bridging atom between aluminum atoms for the formation of oxyfluoroaluminate species, with the presence of Al–O–Al bonds in the bath with low alumina content in the melt, less than 3.5%, or the presence of $\mathrm{Al}{<}_{O}^{O}>\mathrm{Al}$ at high alumina concentrations.^16^ These findings have been proposed by Lacassagne et al.,^17^ using high-temperature NMR techniques, and by Gilbert et al.,^18,19^ based on Raman spectroscopy, with the presence of Al_2_OF_6_^2–^ in low alumina content. Concurrently, Al_2_O_2_F_4_^2–^ is the predominant species at high alumina percentages. In the NaF–AlF_3_–Al_2_O_3_ system, DFT calculations by Picard et al.^20^ suggest the presence of AlOF_2_^–^, Al_2_OF_6_^2–^, and Al_2_O_2_F_4_^2–^, while recent work by Machado et al.^16^ proposes the existence of Al_2_OF_6_^2–^, Al_2_OF_7_^3–^, Al_2_OF_8_^4–^, Al_3_OF_11_^4–^, Al_3_OF_12_^5–^, Al_3_O_2_F_8_^3–^, Al_3_O_2_F_6_^4–^, and Al_4_O_2_F_13_^5–^, with the presence of these complexes increasing accordingly with the content of alumina in the melt. Lin et al.,^21^ using in situ Raman spectroscopy, suggest that the oxygen-containing species in the melt, for various molar ratios and alumina content, are Al_2_OF_4_, Al_3_O_2_F_8_^3–^, Al_2_OF_8_^4–^, and Al_2_O_2_F_4_^4–^, while Zhang et al.^22^ indicate the presence of Al_2_OF_6_^2–^ with alumina content less than 3.5 wt % and Al_2_O_2_F_4_^2–^ for a higher percentage. Furthermore, Chen et al.^23^ investigate the behavior of molten cryolite with calcium fluoride using AIMD simulations. Their results show that the Ca^2+^ ions compete with Al^3+^ for F^–^ and as a result, Ca^2+^ attracts [AlF_*x*_]^(*x*−3)–^ complexes, with preferential cross-linking with AlF_5_^2–^.

In this study, we examine the structural properties of cryolitic melts using AIMD simulations. Computational techniques are employed to investigate the atomic structure and charge distribution within these melts. By focusing on the interactions between aluminum atoms and their surrounding environment, as well as the role of bridging anions, we aim to gain a deeper understanding of the behavior of these complex molten systems at temperatures slightly above the liquidus point.

## 2 Methods

### 2.1 Ab Initio Molecular Dynamics Simulations

Ab initio molecular dynamics (AIMD) simulations were performed using the CP2K 2.6.2 ^24^ software package employing the Quickstep method, which is based on the Gaussian and Plane Wave (GPW) method. This approach combines the efficiency of Gaussian-type orbintals for wave function representation with a plane-wave expansion of the electronic density. To describe electron exchange-correlation effects, Density Functional Theory (DFT) was used with the Perdew–Burke–Ernzerhof (PBE) functional within the Generalized Gradient Approximation (GGA). To account for long-range van der Waals interactions, Grimme’s DFT-D3 dispersion correction was applied.

Core electrons were treated using Goedecker–Teter–Hutter (GTH) norm-conserving pseudopotentials, and valence electrons were represented with the MOLOPT-SR Double-Zeta Valence Polarization (DZVP) basis sets. This combination of PBE, GTH pseudopotentials, and MOLOPT-SR basis sets ensures a balance between computational efficiency and accuracy. A plane-wave cutoff of 400 Ry was applied for the electronic charge density, and a relative cutoff of 60 Ry was used for the energy and force evaluations. A time step of 0.5 fs was used for the AIMD simulations. Each system was equilibrated for 15 ps, followed by a production run of 50 ps for sampling.^15^

### 2.2 Supercells of Investigated Systems

The canonical ensemble (NVT) was employed for all simulations at temperatures slightly above the liquidus point of the respective cryolite melts, with liquidus temperatures calculated using the FactSage 7.0 ^25^ software. The Nosé–Hoover thermostat was used to control temperatures to match the structural dynamics of each system, allowing for the observation of the effects of adding AlF_3_, Al_2_O_3_, and CaF_2_ to the molten cryolite, as shown in Tables 1 and 2. Following industrial practice, the cryolitic bath consisted of cryolite with excess aluminum fluoride, alumina, and calcium fluoride. In this study, a representative cryolitic bath composition was selected: 10 wt % AlF_3_, 3 wt % Al_2_O_3_, and 4.5 wt % CaF_2_. For each system, one component was incrementally added to molten cryolite to reach the target chemical composition. The addition of new components altered the liquidus temperature and the kinetic energy of the melt. To ensure that the systems remained in the liquid state, all simulations were conducted at temperatures slightly above their respective liquidus points.

**Table 1:** Investigated Systems with Calculated Values of Density and % wt Percentage

System	Name	Density_Factsage_ (g/cm^3^)	Density_Kvade H_ (g/cm^3^)	Chemical composition
1	Cryolite	2.095	2.099	100 wt % Na_3_AlF_6_
2	AlF_3_	2.054	2.048	90.09 wt % Na_3_AlF_6_
				9.91 wt % AlF_3_
3	Alumina	2.034	2.036	87.03 wt % Na_3_AlF_6_
				9.95 wt % AlF_3_
				3.02 wt % Al_2_O_3_
4	Bath	2.078	2.070	82.5 wt % Na_3_AlF_6_
				9.90 wt % AlF_3_
				3.00 wt % Al_2_O_3_
				4.6 wt % CaF_2_

**Table 2:** Compositions and Simulation Conditions of the Investigated Molten Cryolite Systems

Name	Al atoms	F atoms	Na atoms	O atoms	Ca atoms	Volume (Å^3^)	Temperature (K)
Cryolite	40	240	120	0	0	6670.15	1285
AlF_3_	46	267	129	0	0	7489.27	1270
Alumina	60	288	126	9	0	8266.91	1255
Bath	49	261	120	9	6	7384.12	1240

The initial atomic configurations were generated by randomly placing atoms within the supercell using the Packmol software.^26^ The volume of each system was determined based on estimated densities derived from thermochemical data, calculated using FactSage 7.0 ^25^ software. These values were found to be consistent with empirical density estmates obtained from Kvande’s equation.^36^ For structural and electronic distribution analyses, radial distribution functions (RDFs) were computed, and visualizations were performed using VMD^28^ and Ovito^29^ software packages.

### 2.3 Structure Analysis

Structural analysis was conducted using RDFs, which describe the probability of finding a given atom at a distance of *r* from a reference atom. The first peak of the RDF provides information about the bond distance between the two atoms. For cryolite melts containing more than 3% w/w alumina, Al_*x*_OF_*y*_ species are more prevalent than Al_*x*_O_2_F_*y*_. When alumina is added to a cryolitic melt with a cryolite ratio (CR) below 3, the focus is on the Al–F bond distance across the full CR range. However, in systems with higher alumina content, such as samples 3 and 4, the key bond distances of interest are O–Al and O–F. Bond distances involving Al or alkaline elements in the cryolitic bath give insight into the melt’s structure. Elements such as sodium and calcium are distributed in the volume of the melt and act as bridges for electrical conductivity and electron movement.

The peak of the normalized RDF function *g*(*r*) indicates the most probable interatomic distance and reflects both chemical bonding and coordination environments. An atom is considered coordinated to a central atom if it lies within a cutoff distance defined by the first minimum following the RDF’s first peak. The coordination analysis is, therefore, crucial to understanding the preferred molecular structures in the cryolite melt. All systems were analyzed after a 15 ps equilibration step and were based on the calculated Mean Squared Displacement (MSD) analysis, which estimated the average distance squared by the current position from the initial position. System equilibration was confirmed when the log­(MSD) versus log­(time) reached a slope of approximately 1, as shown in Figure 1.^27^

**Figure 1:**
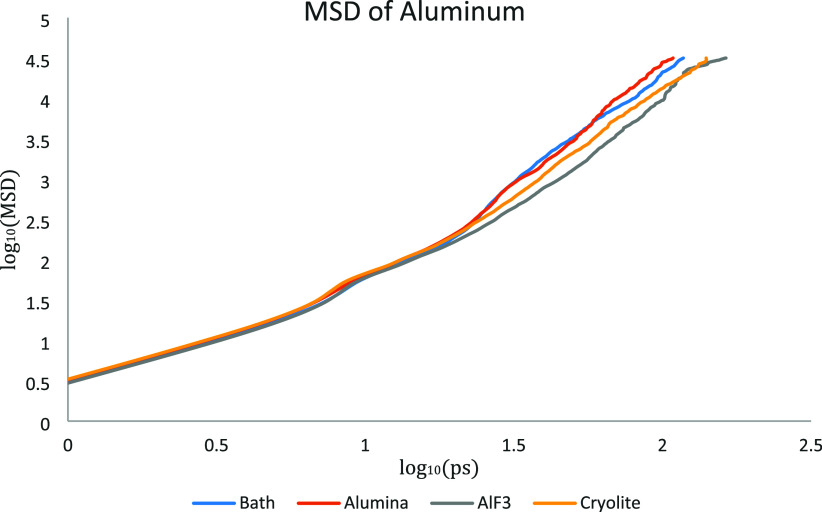
Mean Squared Displacements (MSD) of Al for each system examined (see Table 2).

### 2.4 Electronic Distribution

The total electron density derived from the overlap of atomic orbitals allows for the calculation of partial atomic charges and the assessment of charge transfer phenomena.

The electronic structure and charge distribution in the investigated systems were analyzed by using Mulliken population analysis. This method highlights differences in electronic structure and charge distribution across the molten cryolite systems, including components such as Na_3_AlF_6_, AlF_3_, Al_2_O_3_, and CaF_2_. The insights gained from this analysis help clarify properties such as electrical conductivity and coordination numbers, with a particular focus on the evolution of clusters in the melt and the structural effects of adding or omitting specific substances in the cryolitic bath.

## 3 Results and Discussion

### 3.1 Radial Distribution Function

In this study, aluminum or oxygen atoms were selected as reference atoms for RDF analysis depending on the system investigated. For molten cryolite and the molten Na_3_AlF_6_–AlF_3_ mixture, the aluminum atom is the most critical factor for further investigation of these two systems, using the *g*(*r*) values to understand the basic structure and estimate the melt’s speciation, Figure 2. For cryolite, Table 3, there is a small peak, which indicates cross-linking between the Al-coordinated species^6^ and the intense peak of this bond at 1.75 Å, referring to the distance in the liquid state. The excess of AlF_3_ has a minor effect on the preferred bond distance, as it increases by 0.1 Å for all of the bonds except for F–F, which is located at 2.25 Å, as a result of the increasing number of fluoride atoms in the melt. Although there is minor alteration in the melt’s structure, the bond intensity is more significant than in molten cryolite. Thus, the added AlF_3_ affects strongly the covalent and ionic interactions, mainly the coordination between aluminum atoms and fluoride atoms, and due to the deficient value of the radial distribution factor, it indicates that fluoride ions must cross a significant barrier to exit the first solvation sphere of aluminum ions,^30^ making the Na_3_AlF_6_–AlF_3_ mixture more electronegative than pure cryolite. When alumina dissolves into the mixture, it significantly influences the distance between the Al–Al pair, reducing it to 3.13 Å from 5.75 and 5.86 Å in cryolite and AlF_3_, respectively. The minimization of the Al–Al distance takes place as a result of alumina molecules in the first part and since the creation of oxyfluoroaluminate anions, with low-concentration alumina, less than 3.5%, where oxygen is located at the center of the molecule, creating two bonds with two atoms of aluminum, which are bonded with three atoms of fluoride for each aluminum atom. In the molten mixture of Na_3_AlF_6_–AlF_3_–Al_2_O_3_–CaF_2_, all the preferred distances of the atoms were not affected by the presence of calcium fluoride. However, the resulting molecular structures exhibited different orientations due to the difference in ionic radius between Ca^2+^ and Na^+^ ions, as well as their ability to interact not only with fluoride ions but also with oxygen and aluminum centers. These interactions promote the formation of larger molecular structures, which is in agreement with experimental results showing that the presence of CaF_2_ increased the viscosity of the melt.^35^

**Figure 2:**
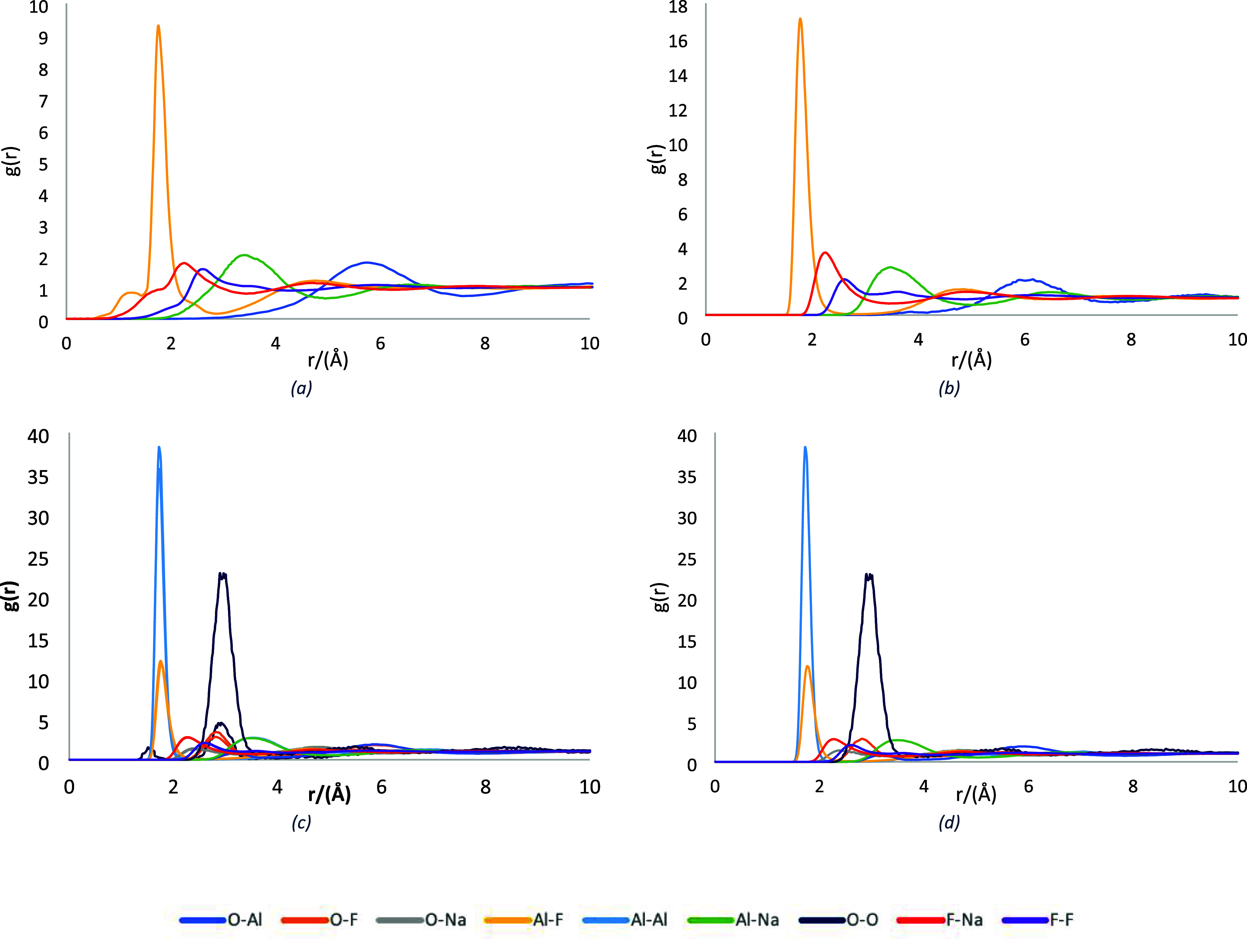
Radial Distribution Function (RDF) of cryolite (a), Na_3_AlF_6_–AlF_3_ (b), Na_3_AlF_6_–AlF_3_–Al_2_O_3_ (c), and cryolitic bath (d) (Na_3_AlF_6_–AlF_3_–Al_2_O_3_–CaF_2_).

**Table 3:** First Peak Distance for Each Pair of Elements

	First Peak distance (Å)			
Pair	Cryolite	Na_3_AlF_6_–AlF_3_	Na_3_AlF_6_–AlF_3_–Al_2_O_3_	Na_3_AlF_6_–AlF_3_–Al_2_O_3_–CaF_2_
Al–Al	5.75	5.86	3.13	3.205
Al–F	1.75	1.78	1.76	1.78
Al–Na	3.38	3.49	3.62	3.48
F–F	2.65	2.60	2.60	2.61
F–Na	2.27	2.24	2.30	2.27
O–Al			1.73	1.74
O–F			2.81	2.81
O–Na			2.42	2.41
O–O			2.95	2.92

### 3.2 Coordination Analysis

All of the trajectories are analyzed for further structural analysis based on the molecules in the melt and the estimation of the coordination number of molecules added for each system, from cryolite to the mixture of the cryolitic bath.

There are free sodium atoms in molten cryolite, Figure 3, while fluoride atoms create complex molecules with aluminum. The AlF_5_^2–^ complex is the predominant species in the melt, followed by four-coordinated aluminum species, as shown in Table 4. An excess of aluminum fluoride is added to the molten cryolite, Figure 4, although AlF_5_^2–^ is retained as the molecule with the highest percentage in the melt, Table 5, and AlF_6_^3–^ has a greater presence in the melt. Simultaneously, the AlF_3_ and AlF_4_^–^ rate decreased.

**Figure 3:**
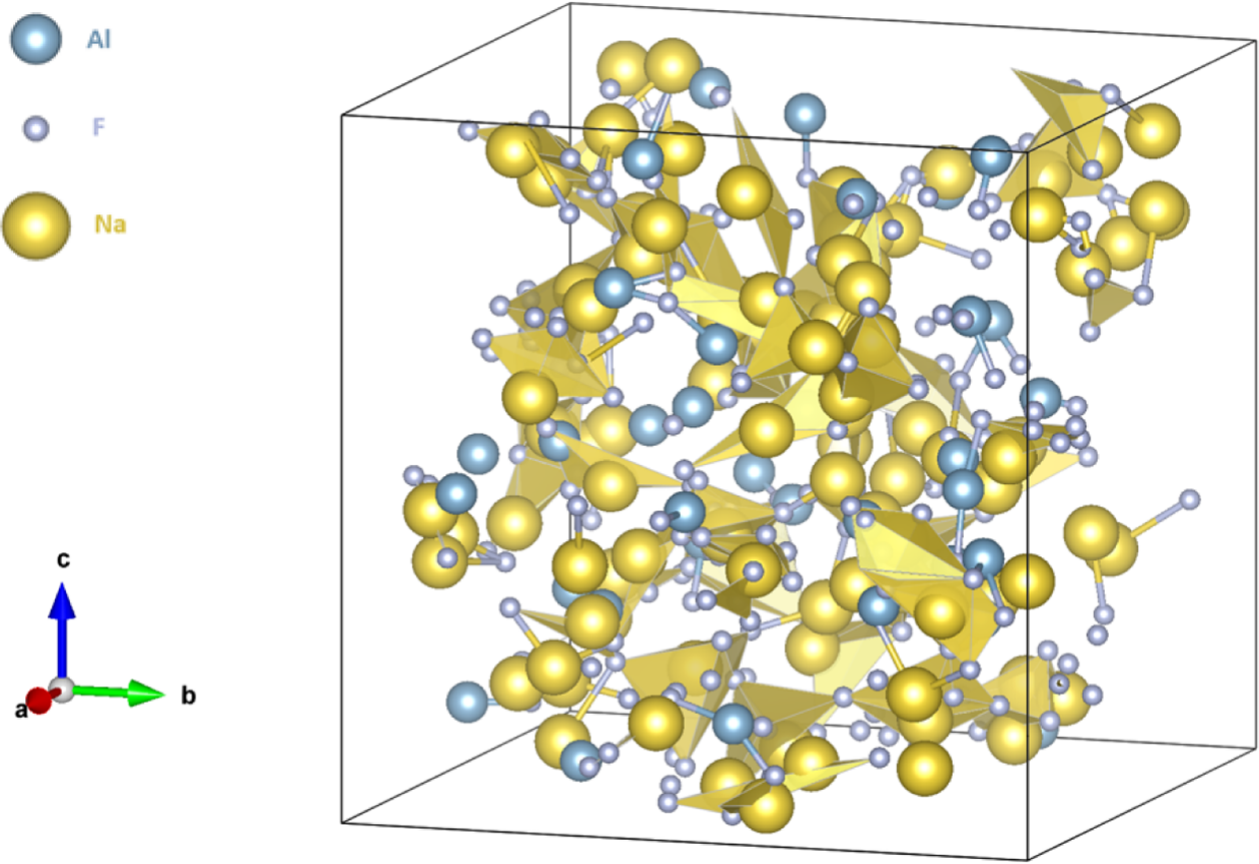
Illustrative representation of molten cryolite.

**Table 4:** Percentages of AlF_3_ and Complex Anions in Molten Cryolite

Molecule	Percentage (%)
AlF_3_	13.99
AlF_4_^–^	19.11
AlF_5_^2–^	46.27
AlF_6_^3–^	13.77

**Table 5:** Percentages of AlF_3_ and Complex Anions in Molten Na_3_AlF_6_–AlF_3_

Molecule	Percentage (%)
AlF_3_	11.59
AlF_4_^–^	17.32
AlF_5_^2–^	46.56
AlF_6_^3–^	19.50

**Figure 4:**
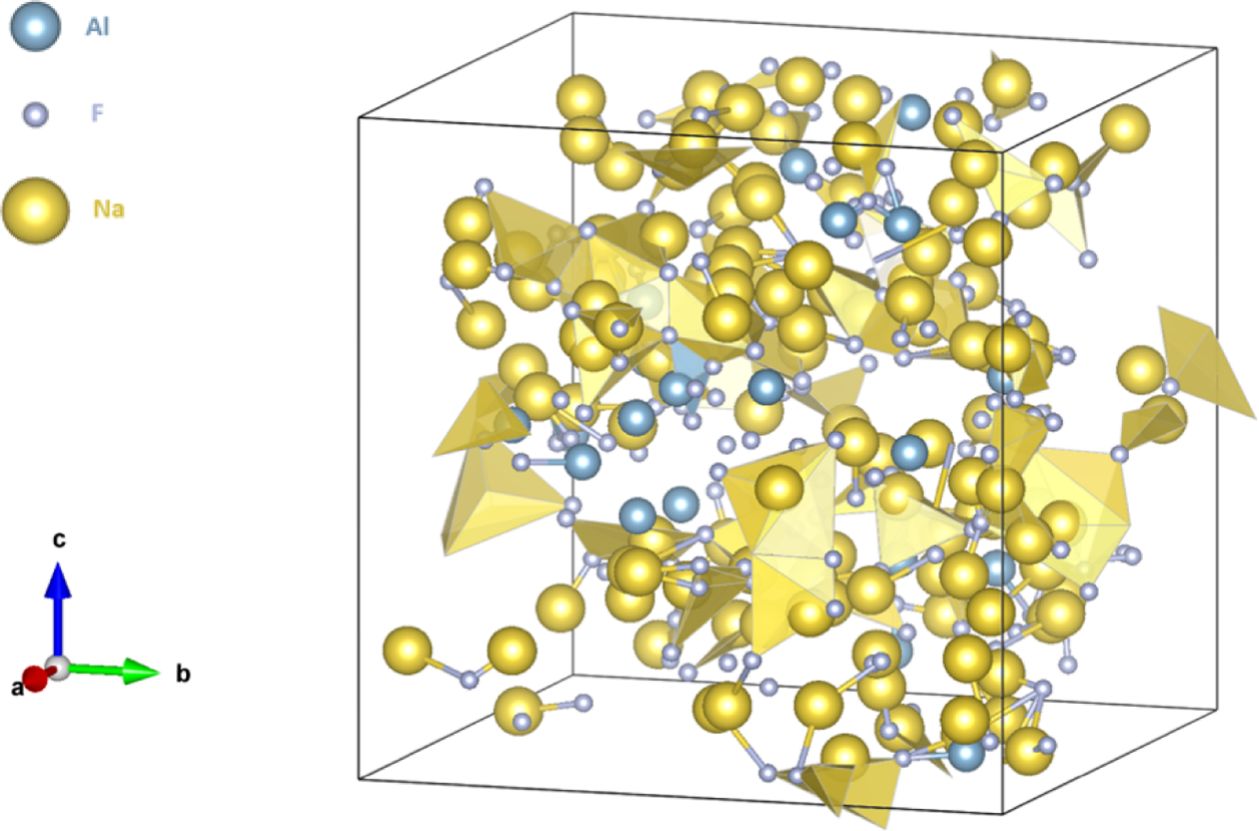
Illustrative representation of molten Na_3_AlF_6_–AlF_3_.

Numerous investigations^31,32^ have shown that if the percentage of alumina is more than 4%, the preferred complex of the system is molecules with two atoms of oxygen in the oxyfluoroaluminate molecules. On the other hand, one oxygen atom creates bonds with aluminum and fluoride with less than 4% of alumina content in the melt. This is in agreement with previous spectroscopic and computational studies,^16–22^ which demonstrate that the structure of the melt evolves depending on alumina addition.

The added alumina in the cryolite mixture with an excess of AlF_3_, Figure 5, leads to the formation of both single and double oxygen aluminum complexes. From the melt’s structure analysis, species such as Al_2_OF_6_^2–^ and Al_2_OF_7_^–^ are identified, while most of the complex ions in the melt consist of AlF_5_^2–^, Table 6. As the percentage of alumina in the melt is 3.5 wt %, there are a few molecules of Al_2_O_2_F_5_^3–^, suggesting the onset of a structural transition from low alumina to high alumina concentration in the cryolitic melt. The presence of such bridging oxygen species has been associated in the literature with enhanced alumina solubility and improved ion transport,^17–21^ which are critical for sustaining steady electrolysis in industrial Hall–Héroult cells.

**Figure 5:**
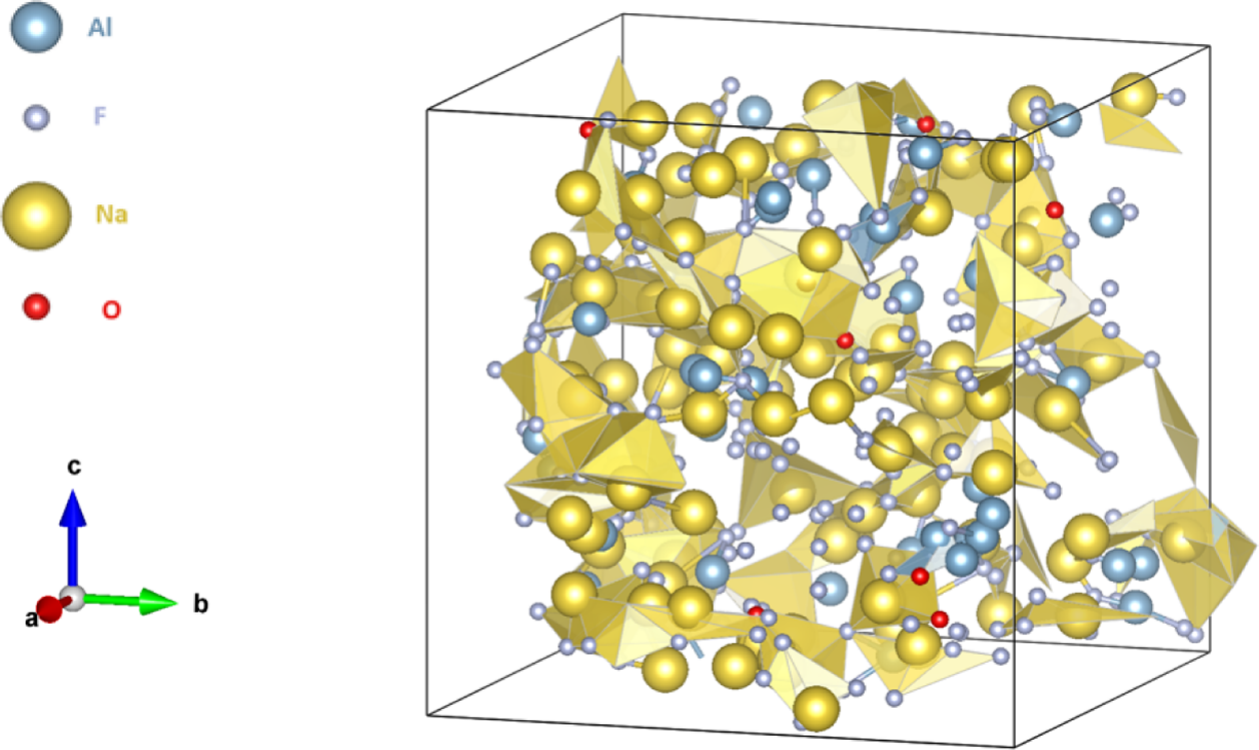
Illustrative representation of molten Na_3_AlF_6_–AlF_3_–Al_2_O_3_.

**Table 6:** Percentages of AlF_3_ and Complex Anions in Molten Na_3_AlF_6_–AlF_3_–Al_2_O_3_

Molecule	Percentage (%)
AlF_3_	20.37
AlF_4_^–^	20.20
AlF_5_^2–^	27.25
AlF_6_^3–^	9.37
Al_2_OF_6_^2–^	1.72
Al_2_OF_7_^3–^	1.36
Al_2_OF_5_^–^	1.16
Al_2_O_2_F_5_^3–^	0.79

In the cryolitic path, a mixture consisting of Na_3_AlF_6_–AlF_3_–Al_2_O_3_–CaF_2_, with calcium fluoride present at 4.5% w/w, affects the conformation of the atoms in the melt, Figure 6. The strong anionic nature of calcium also affects the conformation of molecules in the melt, resulting in an increasing percentage of AlF_5_^2–^. This molecule can surround the calcium atoms in the melt. The percentage of oxyfluoroaluminate ions is increased, with Al_2_OF_6_^2–^ retained as the complex with the greatest presence in the melt, Table 7.

**Table 7:** Percentages of Molecules in the Molten Na_3_AlF_6_–AlF_3_–Al_2_O_3_–CaF_2_

Molecule	Percentage (%)
AlF_3_	14.32
AlF_4_^–^	14.08
AlF_5_^2–^	26.56
AlF_6_^3–^	14.32
Al_2_OF_6_^2–^	2.01
Al_2_OF_7_^3–^	1.59
Al_2_OF_5_^–^	1.35
Al_2_O_2_F_5_^3–^	0.93

**Figure 6:**
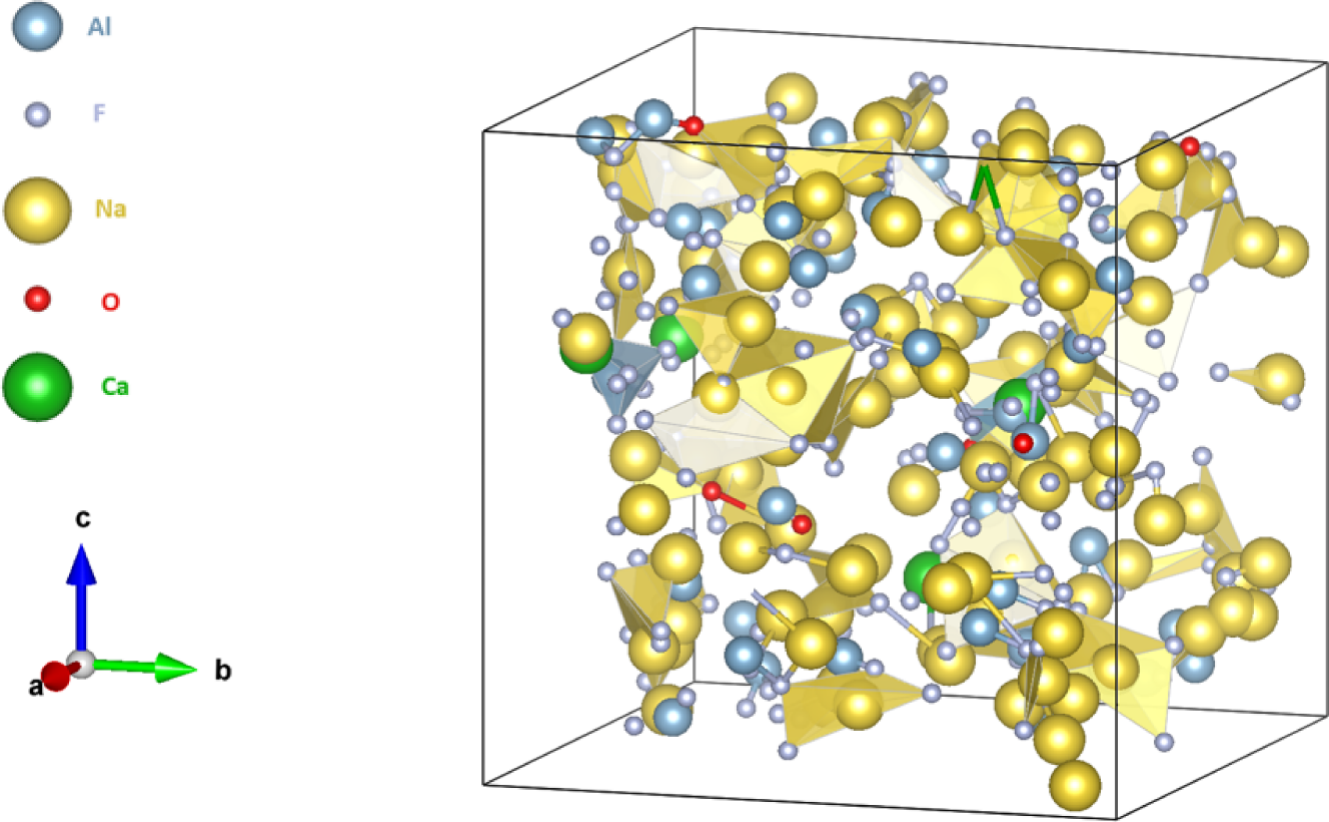
Illustrative representation of molten Na_3_AlF_6_–AlF_3_–Al_2_O_3_–CaF_2_.

### 3.3 Mulliken Analysis

Mulliken population analysis was employed to elucidate the electronic structure and charge distribution in (a) molten cryolite, (b) a mixture of Na_3_AlF_6_–AlF_3_, (c) Na_3_AlF_6_–AlF_3_–Al_2_O_3_, and (d) a cryolitic bath, providing insight into electronic interactions. The results reveal that the addition, respectively, of AlF_3_, Al_2_O_3_, and CaF_2_ to molten cryolite affects the charge distribution in the melt, as shown in Table 8.

**Table 8:** Mulliken Analysis Population for the Investigated Systems

	Average charge distribution (Std)			
Atom	Cryolite	Na_3_AlF_6_–AlF_3_	Na_3_AlF_6_–AlF_3_–Al_2_O_3_	Na_3_AlF_6_–AlF_3_–Al_2_O_3_–CaF_2_
Al	1.17 (0.05)	1.18 (0.06)	1.15 (0.07)	1.14 (0.08)
F	–0.60 (0.08)	–0.64 (0.09)	–0.56 (0.06)	–0.58 (0.07)
Na	0.79 (0.02)	0.78 (0.02)	0.79 (0.02)	0.79 (0.02)
O			–0.69 (0.02)	–0.69 (0.04)
Ca				1.21 (0.05)

Based on the results, the average charge of Al atoms slightly increases after the addition of AlF_3_. However, the presence of alumina and calcium fluoride affects the aluminum atoms’ reversible charge due to the added aluminum atoms from alumina and calcium atoms with more cationic conduction. Fluoride atoms tend to maximize their charge in the presence of an excess of AlF_3_. However, the added alumina minimizes the negative charge, and calcium fluoride affects the charge of fluoride. However, in the cryolite bath, the average charge is less than that in the pure cryolite melt. The average charge of sodium atoms is stabilized at 0.79, except in the mixture of the cryolite bath with aluminum fluoride, in which the average charge of sodium decreases to 0.78. The average charge of oxygen shows a minor increase due to the presence of calcium in the bath, which has the most considerable value among all the atoms in the investigated systems.

## 4 Conclusions

The structure of molten cryolite and its mixtures with alumina has been extensively investigated in the literature through both experimental techniques and molecular dynamics simulations. It is well established that the AlF_5_^2–^ complex is dominant in molten cryolite, followed by AlF_4_^–^ as the second most prevalent, as confirmed by multiple studies and thermodynamic modeling including data from FactSage 7.0. Furthermore, a decrease in the cryolite ratio (CR = NaF/AlF_3_) results in an increased proportion of AlF_4_^–^, while AlF_5_^2–^ remains the primary complex. In this study, based on these established findings, further exploration of cryolite mixtures using AIMD simulations was investigated, with the presence of calcium fluoride and the Mulliken population analysis providing significant information about the structural properties of the cryolite bath.

The addition of alumina at approximately 4 wt % based on industrial practice promotes the formation of oxyfluoroaluminate complexes such as Al_2_OF_6_^2–^ and Al_2_O_2_F_5_^3–^. While the presence of AlF_5_^2–^ is well documented in the literature, Al_2_O_2_F_5_^3–^ appears as a minor complex and becomes more prominent at an alumina content around 3.5 wt % in the melt. In the molten bath, Ca^2+^ ions, due to their higher charges, increase the concentration of AlF_5_^2–^ complexes by reducing the free fluoride anions. In parallel, the percentage of oxyfluoroaluminate complexes increases.

The electronic distribution in these mixtures can emerge using Mulliken population analysis. From a microscopic view of the investigation, it is clear that there are minor changes in the average charge of the atoms, but the total anionic character of the melt increases as the addition of O^2–^ and Ca^2+^ boosts the total charge that has to be equilibrated. The average charge distribution of Al^3+^ ions is influenced by the addition of excess aluminum atoms from alumina. This increase in the anionic character of the melt correlates well with empirical models of electrical conductivity^33^ and aligns with descriptions of the atomic structure.^34^
